# Emergence of carbapenem-resistant enterobacterales co-harboring *bla*_OXA−78_ and *bla*_OXA−58_ from India

**DOI:** 10.1186/s12941-023-00635-6

**Published:** 2023-09-07

**Authors:** Bhaskar Jyoti Das, K. Melson Singha, Jayalaxmi Wangkheimayum, Debadatta Dhar Chanda, Amitabha Bhattacharjee

**Affiliations:** 1 Department of Microbiology, Assam University, Silchar, Dist : Cachar, 788011 Assam India; 2 Department of Microbiology, Silchar Medical College and Hospital, Silchar, Dist : Cachar, Assam, PIN : 788014 India

**Keywords:** Antimicrobial resistance, Carbapenem-resistant Enterobacterales, *bla*_OXA−78_, *bla*_OXA−58_, Tn6080, *ISAba26*

## Abstract

**Background:**

Carbapenem-Resistant Enterobacterales (CRE) has been categorized as pathogens of critical priority by World Health organization (WHO) as they pose significant threat to global public health. Carbapenemase production considered as the principal resistance mechanism against carbapenems and with the recent surge and expansion of carbapenemases and its variants among clinically significant bacteria in India, the present study reports expansion *bla*_OXA−78_ and *bla*_OXA−58_ of in CRE of clinical origin.

**Methods:**

Bacterial isolates were collected from a tertiary referral hospital and identified through VITEK® 2 Compact automated System (Biomerieux, France). Rapidec® Carba NP (Biomerieux, France) was used to investigate carbapenemase production followed by antibiotic susceptibility testing through Kirby-Bauer Disc Diffusion method and agar dilution method. Class D carbapenemase genes were targeted through PCR assay followed by investigation of horizontal transmission of *bla*_OXA−58_ and *bla*_OXA−78_. Whole genome sequencing was carried out using Illumina platform to investigate the genetic context of *bla*_OXA−58_ and *bla*_OXA−78_ genes and further characterization of the CRE isolates.

**Results:**

The carbapenem-resistant *Escherichia coli* (BJD_EC456) and *Serratia marcescens* (BJD_SM81) received during the study from the tertiary referral hospital were isolated from sputum and blood samples respectively. PCR assay followed by whole genome sequencing revealed that the isolates co-harbor *bla*_OXA−58_ and *bla*_OXA−78_, a variant of *bla*_OXA−51_. Horizontal transfer of *bla*_OXA−58_ and *bla*_OXA−78_ genes were unsuccessful as these genes were located on the chromosome of the study isolates. Transposon Tn6080 was linked to *bla*_OXA−78_ in the upstream region while the insertion sequences *ISAba26* and *ISCfr1* were identified in the upstream and downstream region of *bla*_OXA−58_ gene respectively. In addition, both the isolates were co-harboring multiple antibiotic resistance genes conferring clinical resistance towards beta-lactams, aminoglycosides, fluroquinolones, sulphonamides, tetracyclines. BJD_EC180 belonged to ST2437 while BJD_SM81 was of an unknown sequence type. The nucleotide sequences of *bla*_OXA−78_ (OQ533021) and *bla*_OXA−58_ (OQ533022) have been deposited in GenBank.

**Conclusions:**

The study provides a local epidemiological information regarding carbapenem resistance aided by transposon and insertion sequences associated *bla*_OXA−78_ and *bla*_OXA−58_ genes associated and warrants continuous monitoring to prevent their further dissemination into carbapenem non-susceptible strains thereby contributing to carbapenem resistance burden which is currently a global concern.

**Supplementary Information:**

The online version contains supplementary material available at 10.1186/s12941-023-00635-6.

## Background

World Health Organization (WHO) has recognized Carbapenem-Resistant Enterobacterales (CRE) as a significant threat to public health owing to its rate of infection, high mortality rates and widespread transmission potential and categorized them as pathogens of “critical priority” and also has issued guidelines to check their dissemination in healthcare settings [1, 2]. Carbapenemase production is considered as the prime resistance mechanism against carbapenem antibiotics and the genes encoding carbapenemases are usually associated with mobile genetic elements such as plasmids, transposons which helps in their intercellular and intracellular dissemination, maintenance and expression [3, 4].

OXA-78, a variant of OXA-51 has emerged in recent periods within diverse species of Enterobacterales and other non-fermenters [5–7]. Similarly, like OXA-51, its variant *bla*_OXA−78_ gene exhibits weak hydrolytic activity against carbapenems, however, provided a strong transcriptional promoter in the upstream region of the gene associated with mobile genetic elements can contribute to carbapenem resistance thereby compromising therapeutic options [7–9]. In 2005, another class D carbapenemase, *bla*_OXA−58_ was reported in France within a carbapenem-resistant *Acinetobacter baumannii* [10]. The gene was plasmid-borne and the enzyme hydrolyses imipenem, and gradually were reported in pathogens of clinical priority worldwide associated with several outbreaks [11].

Carbapenem resistance determinants aided by diverse mobile genetic elements can confer high level of clinical resistance to carbapenems thereby increasing antibiotic resistance burden which is at present is a global concern. Besides intra and inter specific dissemination; these mobile elements under exposure to selective carbapenem pressure also contributes to the maintenance and expression of carbapenemase genes within bacterial host [3, 4, 12, 13]. With the surge and expansion of carbapenem hydrolyzing class D beta-lactamases (CHDLs) among clinically significant bacteria in India and the paucity of information available; and carbapenems being considered as last therapeutic options against infection caused by multidrug resistant gram-negative bacteria, the present study reports expansion of *bla*_OXA−78_ and *bla*_OXA−58_ in clinical isolates of *Escherichia coli* and *Serratia marcescens*.

## Methods

### Isolates collection and identification

This study was conducted in the Department of Microbiology, Assam University, Silchar. This was part of a DBT, Government of India, funded study for screening of carbapenem non-susceptible Enterobacterales. Among them, two ertapenem non-susceptible Enterobacterales isolates were received in between January and December 2019 from Silchar Medical College and Hospital, a tertiary referral hospital in Silchar, Assam, India. The isolates were recovered from sputum and blood samples of patients admitted to the medicine ward of the tertiary referral hospital. The demographic details of the samples are given in supplementary table S1. The isolates were identified at the species level by VITEK® 2 Compact automated System (Biomerieux, France) and were investigated for carbapenemase production *via* Rapidec® Carba NP (Biomerieux, France) as per manufacturer’s instructions using *Escherichia coli* ATCC 25922 as negative control.

### Antibiotic susceptibility testing

The antimicrobial susceptibility of the two investigated isolates were tested according to the Clinical Laboratory Standard Institute guidelines, CLSI (M100-S32, 2022) recommendations using *Escherichiacoli* ATCC 25922 as quality control strain [14]. The investigated isolates were tested against the following antimicrobial agents, viz., ampicillin (30 µg), cefepime (30 µg), ceftriaxone (30 µg), cefotaxime (30 µg), ceftazidime (30 µg), aztreonam (30 µg), ertapenem (10 µg), imipenem (10 µg), meropenem (10 µg), amikacin (10 µg), gentamicin (10 µg) and ciprofloxacin (5 µg) (HiMedia, India) *via* Kirby-Bauer disc diffusion method. The minimal inhibitory concentrations (MICs) of ertapenem (MSD, France), imipenem (Merck, France) and meropenem (AstraZeneca, UK) were determined through agar dilution method (concentration range : 1–64 µg/ml).

### Molecular detection of class D carbapenemases

Total DNA was extracted from the isolates using boiling-centrifugation method [15]. The presence of class D carbapenemase genes, namely *bla*_OXA−23_, *bla*_OXA−48_, *bla*_OXA−51_ and *bla*_OXA−58_ were detected through PCR assay using previously described primers (Table 1) and reaction conditions and the amplified products were confirmed by sequencing [10, 16–19]. PCR assay was performed in Veriti™ 96-Well Fast Thermal Cycler (Applied Biosystems™, USA) with each single reaction volume of 25 µl containing 2 µl of template DNA (~ 100 ng/µl), 1 µl of each primer (10 pmol/µl), 12.5 µl of 2X GoTaq® Green Master Mix (Promega, Madison, USA) and nuclease free water.

**Table 1:** List of oligonucleotide sequences used as primers for amplification of class D carbapenemase genes in the study

Targeted gene	Primer pairs	5^/^-Sequences-3^/^	Amplified product length (bp)	Reference
*bla*_OXA−23_	OXA-23 F	**5**^**/**^**-**GATCGGATTGGAGAACCAGA**-3**^**/**^	501	16
	OXA-23 R	**5**^**/**^**-**ATTTCTGACCGCATTTCCAT**-3**^**/**^		
*bla*_OXA−48_	OXA-48 F	**5**^**/**^**-**GATTATCGGAATGCCTGCGG**-3**^**/**^	845	17
	OXA-48 R	**5**^**/**^**-**CTACAAGCGCATCGAGCATCA**-3**^**/**^		
*bla*_OXA−51_	OXA-51 F	**5**^**/**^**-**TAATGCTTTGATCGGCCTTG**-3**^**/**^	353	16
	OXA-51 R	**5**^**/**^**-**TGGATTGCACTTCATCTTGG**-3**^**/**^		
*bla*_OXA−58_	OXA-58 F	**5**^**/**^**-**CGATCAGAATGTTCAAGCGC**-3**^**/**^	528	10
	OXA-58 R	**5**^**/**^**-**ACGATTCTCCCCTCTGCGC**-3**^**/**^		

### Horizontal gene transferability assay of ***bla***_OXA−78_ and ***bla***_OXA−58_

To assess the genetic location of *bla*_OXA−78_ and *bla*_OXA−58_ in the genome, transformation and conjugation assays were performed. Plasmids were extracted using QIAprep Spin Miniprep Kit (Qiagen, Germany) as per manufacturer’s instructions and were transformed into recipient strain *Escherichia coli* DH5α by heat shock method and transformants were selected on Luria Bertani agar (HiMedia, India) supplemented with 0.5 µg/ml of imipenem (Merck, France) [20]. For conjugation assay, an azide-resistant *Escherichia coli* J53 was used as recipient strain and transconjugants were selected on Luria Bertani agar (HiMedia, India) medium supplemented with a combination of imipenem (0.5 µg/ml) and sodium azide (100 µg/ml) [21].

### Whole genome sequencing and assembly

Whole genome sequencing was carried out using Illumina platform (outsourced to Bionivid Technology Private Limited, Bengaluru, India). Quality control and data filtering was done using Fastp version 0.20.0 with standard parameters [22]. De novo assembly and scaffolding after quality trimming of the reads was conducted using SPAdes version 3.13.0 [23]. The 16s rRNA gene sequence was predicted using Metaerg version 1.2.0 tool and the nearest genome reference was identified using NCBI BLAST tool (https://www.ncbi.nlm.nih.gov/tools/primer-blast/). Genomes were oriented and rearranged using web-based tool MeDuSa using default web-interface parameters [24]. Genomes were annotated using Prokka version 1.11.1 software [25]. Antimicrobial resistance genes were identified through ResFinder 4.1 (https://cge.food.dtu.dk/services/ResFinder/). Additionally, mobile genetic elements and their relation to resistance determinants were identified through MobileElementFinder version 1.0.3 (https://cge.food.dtu.dk/services/MobileElementFinder/) while plasmids and their possible location in the bacterial genome were screened using NCBI BLAST tool (https://blast.ncbi.nlm.nih.gov/Blast.cgi). PathogenFinder 1.1 was used for finding pathogenicity of the isolates towards human hosts (https://cge.food.dtu.dk/services/PathogenFinder/).

## Results

**Fig. 1:**
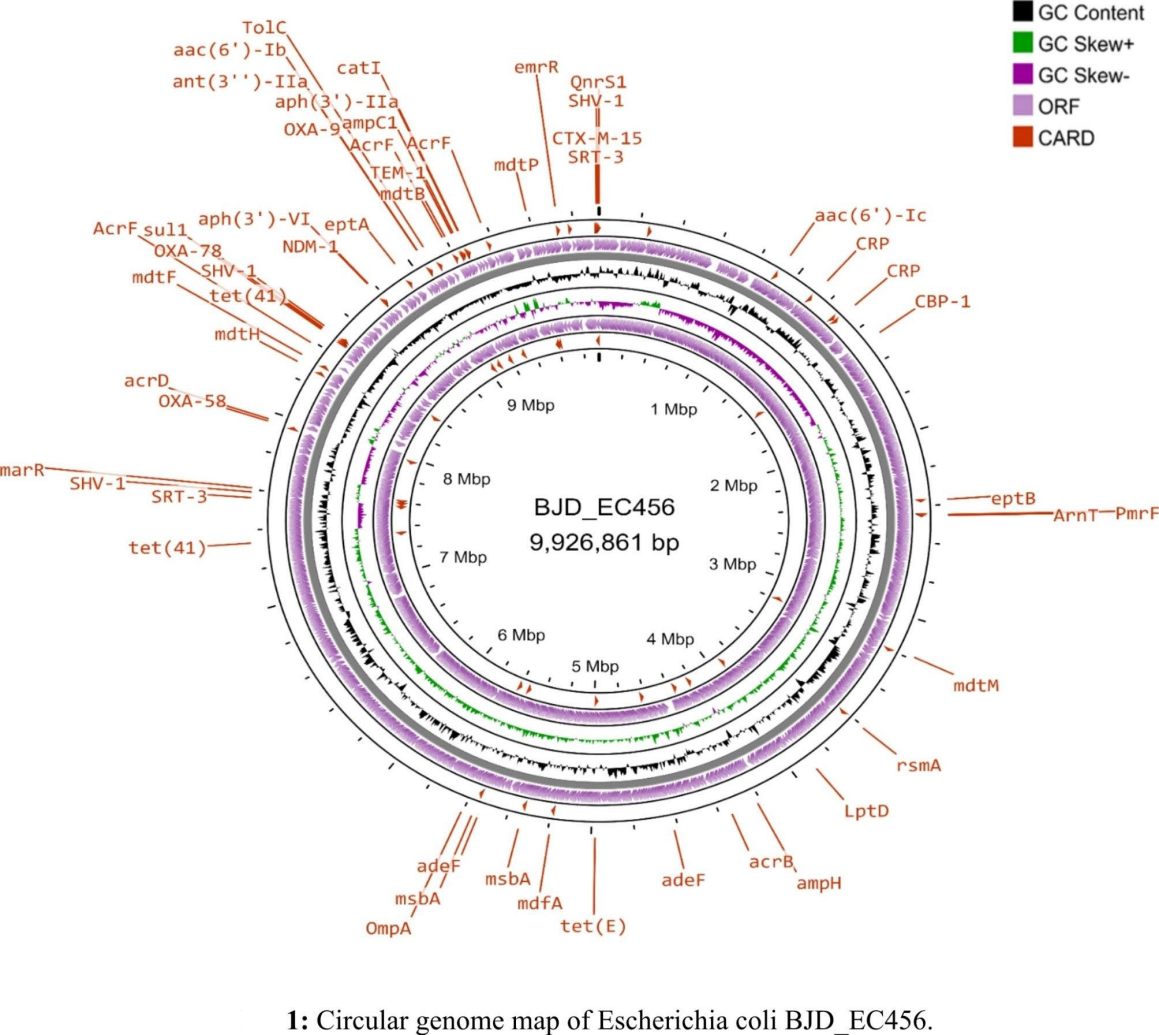
Circular genome map of *Escherichia coli* BJD_EC456. The scale indicates the location in Mbp (chromosome), starting with the initial coding region. The inner and outermost circles represent the backward and forward strands illustrating the coding sequences. The second and third circles shows the GC skew and GC content respectively

**Fig. 2:**
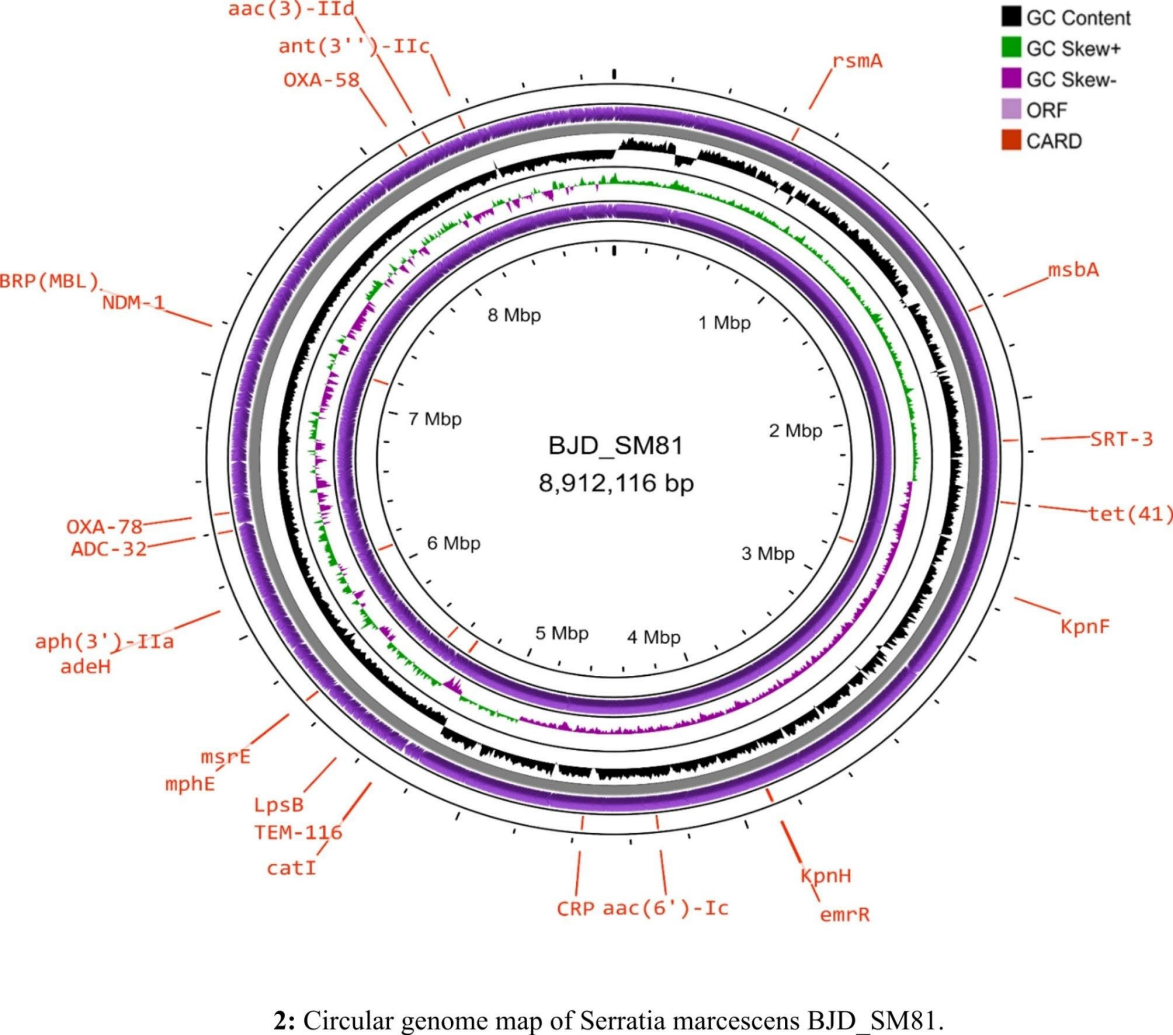
Circular genome map of *Serratia marcescens* BJD_SM81. The scale indicates the location in Mbp (chromosome), starting with the initial coding region. The inner and outermost circles represent the backward and forward strands illustrating the coding sequences. The second and third circles shows the GC skew and GC content respectively

*Escherichia coli* (BJD_EC456) was isolated in 24.01.2019 from sputum sample of a female patient while *Serratia marcescens* (BJD_SM81) was isolated in 27.12.2019 from blood sample of a male patient and both the specimen were collected from the medicine ward of the tertiary referral hospital. Both the isolates were co-harboring *bla*_OXA−78_ and *bla*_OXA−58_ genes and were resistant to all the tested antibiotics and were having MIC above breakpoints (≥ 32 µg/ml) for carbapenems (Table 2). Attempt to transfer the class D carbapenemase genes *bla*_OXA−78_ and *bla*_OXA−58_ from BJD_EC456 and BJD_SM81 by transformation and conjugation was not successful. Whole genome sequenced data revealed that these class D carbapenemase genes were chromosomally located in both the isolates and were associated with mobile genetic elements which might have helped in their acquisition and integration in the bacterial genome. Transposon Tn6080 was associated with the carriage of *bla*_OXA−78_ gene. In case of *bla*_OXA−58_, two insertion sequences were identified in the upstream and downstream region of the gene, IS*Aba26* in the upstream region while in the downstream region IS*Cfr1* was present. Additionally, BJD_EC456 co-harbored multiple resistance genes, such as beta-lactamase genes; *bla*_NDM−1_, *bla*_CTX−M−15_, *bla*_OXA−9_, *bla*_SHV−59_, *bla*_TEM−1_, *bla*_SST−1_, aminoglycoside resistance genes; *aph(3’)-VI*, *aph(3’)-IIa*, *aac(6’)-Ib*, *aac(6’)-Ic*, *aac(6’)-Ib-cr*, *aadA1*, fosfomycin resistance gene; *fosA*, chloramphenicol resistance gene; *catA1*, quinolone resistance genes; *qnrS1*, sulphonamides resistance gene; *sul1*, tetracycline resistance gene; *tet*(41) and antiseptic resistance gene; *qacE*, along with five plasmids viz. Col440I, IncFII(pKPX1), IncFIB(K), IncFIB(pKPHS1) and IncM1 (Fig. 1). BJD_SM81 contained Col(MG828) plasmid and carried the following resistance genes, such as beta-lactamase genes; *bla*_NDM−1_, *bla*_TEM−116_, *bla*_ADC−25_, *bla*_SST−1_, aminoglycoside resistance genes; *aph(3’)-IIa*, *aac(3)-IId*, *aac(6’)-Ic*, chloramphenicol resistance gene; *catA1*, tetracycline resistance gene; *tet*(41), macrolide resistance genes; *msr(E)*, *mph(E)* and OqxB_1 belonging to RND efflux pump family conferring resistance against various antibiotics, like quinolones, nitrofurantoin, quinoxalines, tigecycline, chloramphenicol, detergents and disinfectants (Fig. 2). Multi Locus Sequence Typing (MLST) results showed that BJD_EC180 belonged to *Escherichia coli* sequence type ST2437, while BJD_SM81 belonged to an unknown sequence type. The nucleotide sequences of *bla*_OXA−58_ and *bla*_OXA−78_ have been deposited in GenBank under the accession numbers OQ533022 and OQ533021 respectively, and the profiles of BJD_SM81 and BJD_EC456 have been summarized in Table 3.

**Table 2:** Antibiogram of BJD_EC456 and BJD_EC81 co-harboring *bla*_OXA−78_ and *bla*_OXA−58_

Isolates ID	Organism	Isolation date	Specimen	Resistance profiles	MICs (µg/ml)		
					ERT	IMP	MEM
BJD_EC456	*Escherichia coli*	24.01.2019	Sputum	AMP, FEP, CRO, CAZ, ATM, ERT, IMP, MEM, AMK, GEN, CIP	≥ 64	≥ 32	≥ 64
BJD_SM81	*Serratia* *marcescens*	27.12.2019	Blood	AMP, FEP, CRO, CAZ, ATM, ERT, IMP, MEM, AMK, GEN, CIP	≥ 64	≥ 32	≥ 64

AK: amikacin, AMP: ampicillin, AT: aztreonam, CPM: cefepime, CTX: Cefotaxime,

CTR: ceftriaxone, CTP: ciprofloxacin, CAZ: ceftazidime, GEN: gentamicin,

ETP: ertapenem, IPM: imipenem, MRP: meropenem

**Table 3:** Profiles of BJD_EC456 and BJD_SM81

Isolates ID	*bla*_OXA−51_ variant	Carbapenemases	β-lactamases	Aminoglycoside resistance genes	Other resistance genes	Virulence genes	Mobile genetic elements	Sequence type
BJD_EC456	*bla*_OXA−78_	*bla*_OXA−58_, *bla*_NDM−1_	*bla*_OXA−9_, *bla*_SHV−59_, *bla*_TEM−1_, *bla*_CTX−M−15_, *bla*_SST−1_	*aph(3’)-VI*, *aph(3’)-IIa*, *aac(6’)-Ib*, *aac(6’)-Ic*, aac(6’)-Ib-cr	*aadA1*, *qacE*, *fosA*, *catA1*, *qnrS1*, *sul1*, *tet*(41)	*nlpl*, *mrkA*, *iutA*, *fimH*, *gad*, *clpK1*	Col440I, IncFII(pKPX1), IncFIB(K), IncFIB(pKPHS1), IncM1, IS*Aba26*, IS*Cfr1I*, Tn6080	ST2437
BJD_SM81	*bla*_OXA−78_	*bla*_OXA−58_, *bla*_NDM−1_,	*bla*_SST−1_, *bla*_TEM−116_, *bla*_ADC−25_	*aph(3’)-IIa*, *aac(3)-IId*, *aac(6’)-Ic*	OqxB_1, *msr(E)*, *mph(E)*, *catA1*, *tet*(41)	*clpK1*	Col(MG828) IS*Aba26*, IS*Cfr1*, Tn6080	Unknown

## Discussion

Carbapenem are the most potent antibiotics among all clinically available beta-lactam antibiotics and are used as last resort drugs to treat infection caused by multidrug resistant Gram-negative bacteria. Over the recent years, with the emergence of CRE has threatened this class of antibiotics and pose a serious threat to global public health. In India also, reports of CRE isolates have been increased significantly over the years [26–29]. In this study, we reported the co-carriage of *bla*_OXA−78_, a variant of *bla*_OXA−51_ and *bla*_OXA−58_ genes in two CRE isolates (*Escherichia coli* and *Serratia marcescens*) obtained from a tertiary referral hospital in northeastern part of India. The finding of our study is in congruence with a recent study conducted in 2022, that reported the co-occurrence of *bla*_OXA−51−like_ and *bla*_OXA−58_ genes in Enterobacterales isolates recovered from urine samples of UTI patients from a hospital of Tehran, Iran [5]. Similarly, study conducted by Leski and his team in 2013 also reported the co-existence of *bla*_OXA−51−like_ and *bla*_OXA−58_ genes within Enterobacterales isolates obtained from Mercy Hospital, Bo, Sierra Leone [6]. In India, the co-carriage of *bla*_OXA−51−like_ and *bla*_OXA−58_ genes was reported in 2015 in carbapenem-resistant *Acinetobacter baumannii* isolated from various clinical specimens obtained from a university teaching hospital [24–26]. However, to the best of our knowledge this is the first report of co-occurrence of *bla*_OXA−78_ and *bla*_OXA−58_ genes in Enterobacterales from India.

In the present study, the chromosomally located *bla*_OXA−78_ and *bla*_OXA−58_ genes were found associated with diverse mobile genetic elements. Tn6080 transposon was observed with the carriage of *bla*_OXA−78_ gene in both the isolates. This finding is in accordance with previous studies that reports this transposon as a carrier of *bla*_OXA−51_ genes and its variants [13, 30, 31]. In case of *bla*_OXA−58_ gene, IS*Aba26*, a single nucleotide variant belonging to the IS*Aba256* family in the upstream region and IS*Cfr1* belonging to IS1182 family in the downward region were found associated with the gene. These carbapenem resistance determinants associated with transposon and insertion sequences possess a serious health hazard as transposition of these mobile genetic elements can alter bacterial gene expression thereby increasing antibiotic resistance burden which is at present is a global concern [13]. These mobile elements facilitate mobilization of carbapenemase genes thereby aiding in their intra and inter specific dissemination; and acquisition of such elements by susceptible phenotypes results into the evolution of resistant ones [3, 4, 13]. Selective pressure induces adaptive response and led to the emergence and expansion of antibiotic resistance thereby contributing to resistance burden which is at present a serious threat to global public health due to its limiting effect on therapeutic options. It is evident from previous studies that insertion sequences play a major role in conferring clinical resistance to carbapenems as their insertion upstream of *bla*_OXA_ genes provides a strong outward promoter thereby aiding in better expression of otherwise silent *bla*_OXA_ genes encoding carbapenemases [13, 32]. Selective antibiotic pressure also contributes in the maintenance of transposon within the host genome that carry antibiotic resistance genes [13]. And in accordance, in our study also it was observed that the *bla*_OXA−78_ and *bla*_OXA−58_ genes were maintained within such unnatural hosts by their respective insertion sequences and transposons and also the isolates co-harboring them exhibited high MICs for carbapenem antibiotics. These findings highlight the role of positive selection pressure generated by the surge in usage of carbapenems within the study center that aid in the maintenance of mobile genetic elements carrying *bla*_OXA−78_ and *bla*_OXA−58_ genes and also in the expression of these resistance determinants conferring clinical resistance to carbapenems, antibiotic of last resort.

Studies suggests that isolates harboring carbapenemase encoding genes often carry additional resistance genes that confer resistance to other beta-lactams, aminoglycosides, fluroquinolones, sulphonamides, tetracyclines and other antibiotics, and in accordance, our study isolates also co-harbored multiple resistance genes elucidating their multidrug resistant nature correlating with the observations of antibiotic susceptibility testing [33, 34]. The extensive usage of carbapenems in clinical settings especially of developing countries is already an established risk factor for emergence of carbapenem-resistant organisms and might also have played a vital role in the maintenance and elevated expression of resistance determinants associated with carbapenem resistance [35]. Carbapenem resistance in Enterobacterales is predominantly associated with the horizontal dissemination of genes encoding carbapenem-hydrolyzing carbapenemase enzymes and therefore, these carbapenem resistance genes are often found associated with mobile genetic elements that aids in their capture, accumulation and intracellular and intercellular dissemination thereby significantly contributing to carbapenem resistance worldwide [4, 36]. Several studies reports the presence of insertion sequences such as IS*Aba1*, IS*Aba2* and IS*Aba3* in both upstream and downstream regions of *bla*_OXA−51_ ana *bla*_OXA−58_ genes and also suggested that insertion sequences located upstream of *bla*_OXA_ genes upregulates the expression of these carbapenemase genes by providing a transcriptional promoter [9, 10, 13, 30, 31, 37–39]. So, far there is no published report of *Escherichia coli* ST2437 harboring *bla*_OXA−78_ and *bla*_OXA−58_ genes or other *Escherichia coli* sequence types with the carriage of these resistance genes. Therefore, the carriage of *bla*_OXA−78_ and *bla*_OXA−58_ genes in this sequence type (ST2437) in the current study is of epidemiological importance. *bla*_OXA−78_ and *bla*_OXA−58_ genes conferring resistance towards carbapenems aided by mobile genetic elements possess a serious health hazard as potential source and vehicle of future dissemination and warrants urgent monitoring as they pose a threat to the control of antimicrobial resistance and endangering our fight against antimicrobial resistance.

## Conclusions

Antimicrobial resistance, at present is a global concern and with the increase in incidence of class D carbapenemases and its variants among clinically significant gram-negative bacteria, the findings of the present study, provide a local epidemiological information regarding carbapenem resistance and mobile genetic elements associated dissemination of *bla*_OXA−78_ and *bla*_OXA−58_ genes in carbapenem-resistant isolates of *Escherichia coli* and *Serratia marcescens* of clinical origin. Since carbapenems are regarded as antibiotic of last resort for the treatment of infections caused by multi-drug resistant gram-negative bacteria, the findings of the present study warrant continuous monitoring of these carbapenem resistance determinants considering their association with mobile genetic elements; along with a scope to design and assess strategies to prevent the spread and emergence of carbapenem resistance determinants and accordingly optimize clinical therapy to avoid treatment failure.

### Electronic supplementary material

Below is the link to the electronic supplementary material.

Supplementary Material 1

## Data Availability

The datasets used and/or analysed during the current study are available from the corresponding author on reasonable request.

## Declarations

### Nucleotide sequence accession number

The nucleotide sequences of *bla*_OXA−58_ and *bla*_OXA−78_ have been deposited in GenBank under the accession numbers OQ533022 and OQ533021 respectively.

## Abbreviations

WHO: World Health Organization
CRE: Carbapenem-Resistant Enterobacterales
CHDLs: Carbapenem Hydrolyzing Class D Beta-lactamases
bla: Beta-lactamases
OXA: Oxacillinase
CLSI: Clinical Laboratory Standard Institute
ATCC: American Type Culture Collection
MIC: Minimum Inhibitory Concentration
DNA: Deoxyribonucleic Acid
ng: Nanogram
µl: Microlitre
pmol: Picomole
µg: Microgram
ml: Millilitre
NCBI: National Center for Biotechnology Information
BLAST: Basic Local Alignment Search Tool
Tn: Transposon
IS: Insertion Sequence
Inc: Incompatibility
MLST: Multi Locus Sequence Typing
ST: Sequence Type
UTI: Urinary Tract Infection
