# Supplementary material for: Emergence of carbapenem-resistant enterobacterales co-harboring blaOXA−78 and blaOXA−58 from India
Source: Ann Clin Microbiol Antimicrob. 2023 Sep 7;22:79. doi: 10.1186/s12941-023-00635-6 (PMC10486080; doi:10.1186/s12941-023-00635-6)
Supplement: Supplementary file 1 — Supplementary Material 1 [file 12941_2023_635_MOESM1_ESM.docx]

**Supplementary table S1: Clinical and demographic details of the isolates used in the study**

| **Sample ID** | BJD_EC456 | BJD_SM81 |
| --- | --- | --- |
| **Organism** | *Escherichia coli* | *Serratia* *marcescens* |
| **Date of Isolation** | 24.01.2019 | 27.12.2019 |
| **Specimen** | Sputum | Blood |
| **Ward** | Medicine | Medicine |
| **Patient’s Gender** | Female | Male |
| **Patient’s** **Age** | 65 | 37 |
| **Region** | Hilakandi, Assam, India  24°40'48.00" N  92°34'12.00" E | Karimganj, Assam, India  24° 51' 53.6904'' N  92° 21' 32.9544'' E |
